# Architectural Glazed Tiles Used in Ancient Chinese Screen Walls (15th–18th Century AD): Ceramic Technology, Decay Process and Conservation

**DOI:** 10.3390/ma14237146

**Published:** 2021-11-24

**Authors:** Jingyi Shen, Li Li, Ji-Peng Wang, Xiaoxi Li, Dandan Zhang, Juan Ji, Ji-Yuan Luan

**Affiliations:** 1 School of History and Culture, Shandong University, Jinan 250100, China; jingyi.shen@sdu.edu.cn (J.S.); echo_zhangdandan@126.com (D.Z.); 2 Shaanxi Institute for the Preservation of Cultural Heritage, Xi’an 710075, China; jijuan107@163.com; 3 School of Civil Engineering, Shandong University, Jinan 250012, China; Jasperluan@outlook.com; 4 Department of Conservation, Emperor Qinshihuang’s Mausoleum Site Museum, Xi’an 710600, China; riverlx2@126.com

**Keywords:** glazed tile, screen wall, ceramic technology, decay, conservation, ancient China

## Abstract

The glazed tile is an important building material used throughout the history of traditional Chinese architecture. Architectural glazed tiles used to decorate the screen walls of ancient China are studied scientifically for the first time. More than 30 glazed tile samples from the screen walls of the 15th to 18th century AD of the Hancheng Confucian Temple and Town God’s Temple in Shaanxi Province were carefully investigated using SEM–EDS and XRD. Microstructure and chemistry indicated the raw materials, the recipes and the technological choices used to produce the paste and glaze of the glazed tile samples studied. The causes for the key degradation processes of these glazed tiles used as building materials in the screen walls have also been discussed. This work has clear implications for the restoration and conservation treatments on these kinds of ancient Chinese building materials.

## 1. Introduction

### 1.1. Chinese Glazed Tiles

**Figure 1:**
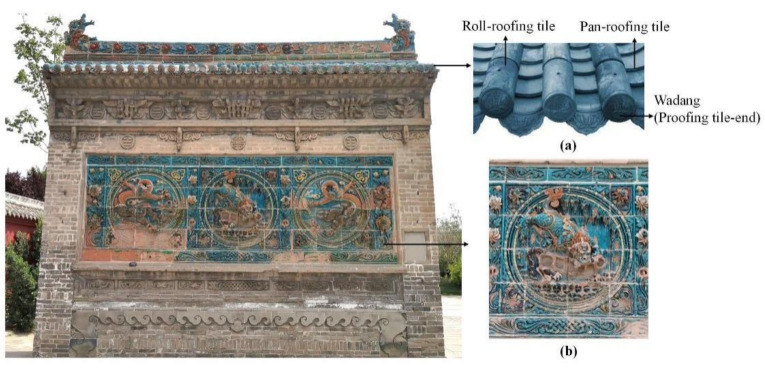
Glazed tiles decorated in different positions of ancient Chinese architecture. (This picture is the South screen wall in the Hancheng Confucian Temple) (**a**) Glazed tiles decorated on the roof. (**b**) Glazed tiles decorated on a screen wall.

The glazed tile is an important building material used throughout the history of traditional Chinese architecture due to its artistic appearance and waterproof qualities. Additionally, glazed tiles were widely used in ancient top-tier Chinese architectural structures such as royal palaces, mausoleums, imperial gardens and temples as a significant part of ancient architectural etiquette. Architectural glazed tiles were used to decorate imperial palaces in China as far back in history as the Northern Wei Dynasty (386–534 AD) [1]. Until the Ming (1368–1644 AD) and Qing (1644–1911 AD) dynasties, the use of architectural glazed tiles grew to the peak period. According to the different positions and functions used in ancient architecture, glazed tiles can be divided into two categories. The first is the glazed tiles used as building materials on the roofs, such as roofing tile-ends (known as Wadang in Chinese), pan-roofing tiles and roll-roofing tiles (Figure 1a). The other kind is glazed tiles decorated on the walls, such as the walls of pagodas and screen walls (known as Yingbi in Chinese) (Figure 1b).

Previous scientific research on Chinese glazed tiles has primarily focused on the following areas: the manufacturing technique of glazed tiles production [2,3,4]; the composition and provenance of their raw materials [5,6] and the main deterioration mechanism and conservation including glaze fading and the shedding of the glaze layer [7,8,9]. However, the glazed tiles that have been studied are mainly from the Beijing city and its surrounding areas, while architectural ceramic tiles from other areas have rarely been studied scientifically. In addition, the majority of previous studies mainly assessed the glazed tiles used on the roofs, whereas studies on the glazed tiles used to decorate the walls of ancient architecture are scarce. The only scientific research conducted to date is a study of the manufacturing technique of glazed tiles used to decorate the walls of the Ming dynasty Baoensi Pagoda located in Nanjing City in South China [10].

Glazed tiles decorated on the roofs and walls are used in different positions of architectures with different shapes. This means that they may have distinct ceramic technology, suffer from different decay processes and have variation in how well they can be preserved. Moreover, these differences may also exist in the glazed tiles produced and preserved in different areas of China. Therefore, it is of great significance to especially study the representative glazed tiles used as ancient building materials to decorate walls, which is crucial for taking effective protection measures for such building materials.

The present paper scientifically studies the Chinese glazed tiles used to decorate ancient screen walls, which is rarely considered in previous studies. Raw materials used, the manufacturing techniques applied and the degradation processes of this kind of glazed tiles in the Hancheng Confucian Temple and Town God’s Temple are discussed in detail for the first time. This research enables us to propose correspondingly helpful suggestions on the preservation and restoration of these tiles.

### 1.2. The Glazed Tiles on the Screen Walls of the Hancheng Confucian Temple and Town God’s Temple Building Complexes

**Figure 2:**
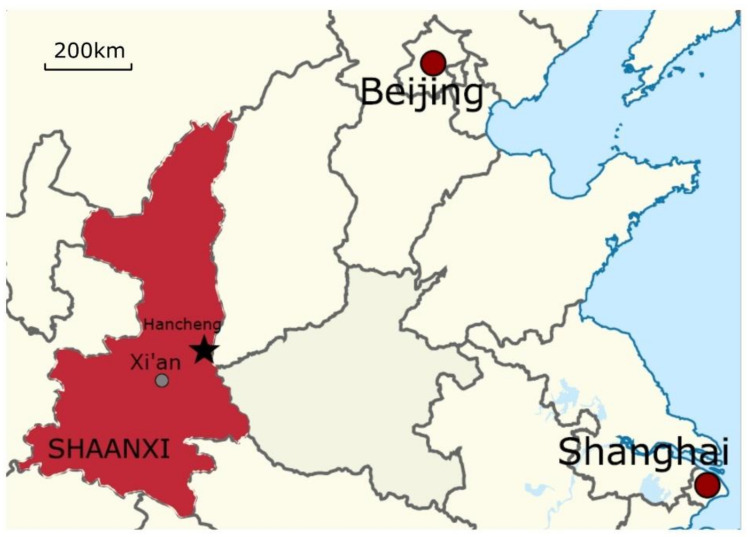
The location of the Hancheng city where the screen walls studied are located.

The screen wall is an isolated standing wall normally built inside the courtyard of a traditional Chinese building complex, or located on each side of the gate, to shield the rest of the buildings from view. This paper focuses on the exquisite glazed tiles which decorate the screen walls of the Hancheng Confucian Temple and the Hancheng Town God’s Temple (known as Chenghuang Miao in Chinese) building complexes in Hancheng City, Shaanxi province. The locations of these buildings are shown in Figure 2.

The Confucian Temple is built in the memory of Confucius, who is the originator of Confucius culture, while the Town God’s Temple is the Taoist temple of the town god. The Hancheng Town God’s Temple is the most integrated Town God’s Temple building complex in Northwest China, and the Hancheng Confucian Temple is the third-largest Confucian temple building complex. Both the Hancheng Confucian Temple and Town God’s Temple building complex have a prestigious reputation because of their exquisite glazed tiles decorated on the screen walls. All these enable them to be concerned as key historical and cultural sites under state protection in China.

**Figure 3:**
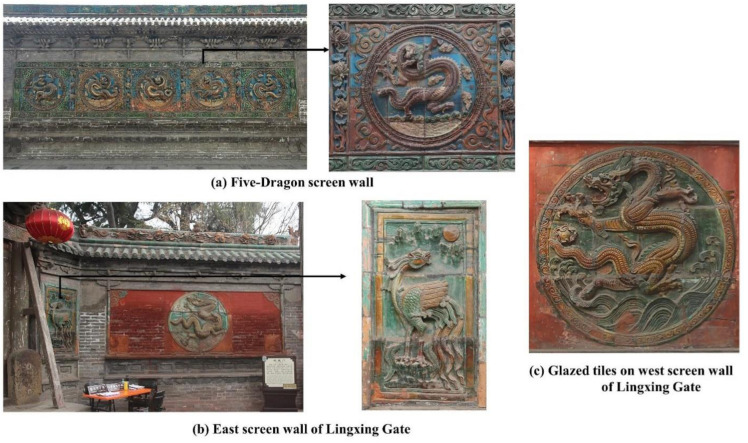
Screen walls with glazed tile decoration in the Hancheng Confucian Temple building complex.

In the Hancheng Confucian Temple building complex, the glazed tile decoration is mainly concentrated on five screen walls comprising a total area of approximately 139.34 m^2^. The main decorative patterns of these glazed tiles are dragons and phoenixes, as well as some flower patterns. All these dragon and phoenix patterns are vivid in colors and shapes, and the total number of dragon patterns is exactly nine, representing the highest level of architectural etiquette. Several screen walls with glazed tiles can be seen in Figure 3.

**Figure 4:**
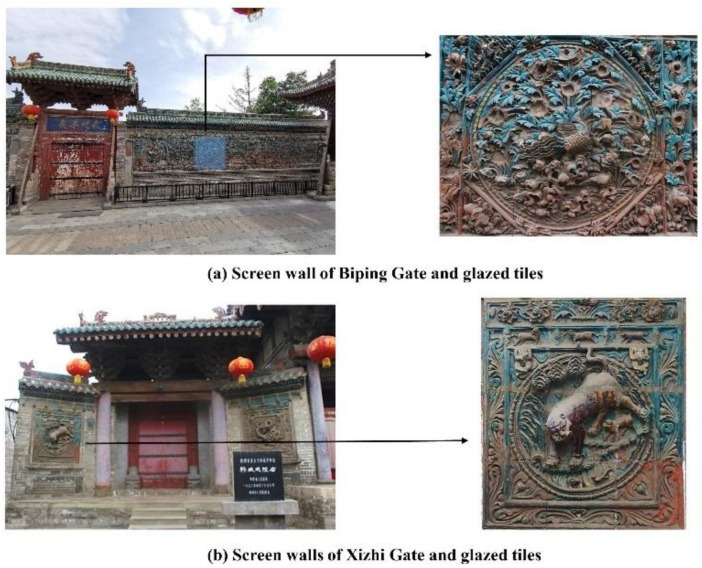
Screen walls with glazed tile decoration in the Hancheng Town God’s Temple building complex.

In the Hancheng Town God’s Temple building complex, the glazed tile decoration is mainly centered on five screen wall buildings with a total area of around 225.34 m^2^. Almost all the screen walls are mainly decorated with glazed tiles of turquoise color, and supplemented by green, yellow and brown glazed tiles which are used to form patterns. The main decorative patterns of these glazed tiles are dragons, phoenixes and tigers, as well as scenery with hills, water, pavilions, flowers and little animals. Several screen walls with glazed tiles can be seen in Figure 4.

## 2. Materials and Methods

### 2.1. Sample Information

**Figure 5:**
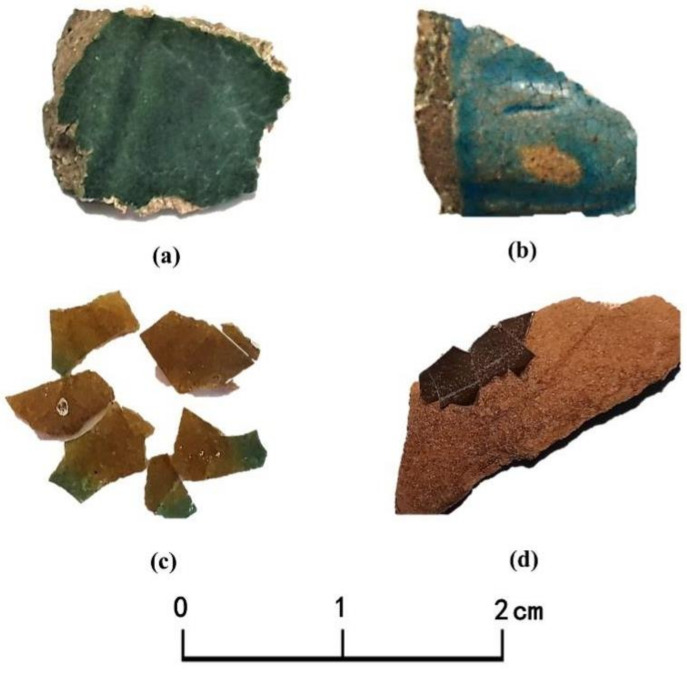
Photographs of the samples representing the four glaze colors in this study. (**a**) Green; (**b**) Turquoise; (**c**). Yellow; (**d**). Brown.

Thirty-three fragments of glazed tiles traced back to several different periods of the Ming and Qing dynasties (ca. 1482–1722 AD) were collected in this study. They include 17 pieces from four different screen walls of the Hancheng Confucian Temple and 16 samples from 4 different screen walls of the Town God’s Temple. The glazed tile samples cover all glaze colors, namely green, yellow, brown and turquoise. Details are shown in Table 1. The appearances of some typical samples are shown in Figure 5.

### 2.2. Analytical Methods

#### 2.2.1. Scanning Electron Microscopy (SEM) with Energy Dispersive Spectroscopy (EDS)

A ZEISS instrument, model EVO-250 SEM (Zeiss, the instrument is located in Xi’an, China), coupled with an Oxford Instruments X-MAX20 EDS system, was used to study the sample’s microstructure and the body and glaze compositions. A cross-section of each representative sample was taken off and mounted as a polished block. The acceleration voltage used for observation and analysis was 20 keV with a working distance of 8 mm. SEM backscattered electron (BSE) images of the microstructure of the body paste and glaze were recorded. For each sample, the chemical compositions of the tile body and glaze were analyzed in three micro areas and the average values were calculated. The size analyzed by EDS both for the body and the glaze is around 200 μm× 300 μm. The test time of EDS was 90 s. Oxford Instruments standards were used to quantify 10 elements (Si, Al, Na, K, Mg, Ca and Fe for the tile body, while Pb, Si, Al, Na, K, Mg, Ca, Fe, Cu and Ti for the tile glaze). The results were converted into oxide percentages and normalized to 100%. The detection limit of the instrument is 0.1%. The glaze chemical compositions of all 33 samples have been analyzed in this study. For the chemical compositions of paste, only 23 samples (C1–10 and T1–13) have been analyzed. The reason is that the pastes of samples C11–17 and samples T14–16 have already decayed heavily, so the pastes of these samples look loose and fragile (having suffered from salt efflorescence); therefore, if these samples were tested for their chemical compositions, inaccurate results would be obtained.

#### 2.2.2. X-ray Diffraction (XRD)

Eighteen tile bodies of the samples and two samples of solute salt powder were tested by XRD (RIGAKU SmartLab) for their phase analysis. Before the test, a small amount of the tile body of each sample was taken off and ground into fine powder. A 2θ range of 5–55° was used, with the detector type being a D/teX Ultra 250 silicon strip detector device (SmartLab, the instrument is located in Xi’an, China). A tube voltage of 30 kV with a current of 300 mA was applied.

#### 2.2.3. Analytical Methods for Degradation Process

To discuss body salt efflorescence, a glazed tile sample (T-15) with intact glaze but weathered-looking paste was scanned by a micro-CT scanner (Nanovoxel 3000, the instrument is located in Tianjing, China) with 190 keV voltage, and a 25 W power was used on the X-ray source. Sequences of grayscale images were obtained, which can be reconstructed into a 3D image. The image resolution is 0.5 μm per voxel length.

The moisture contents at different heights (25 to 250 cm from the ground with 25 cm as the measurement interval) of five screen walls have been tested with the HF SENSOR MOIST 210 non-destructive moisture measurer (HF SENSOR, the instrument is located in Xi’an, China). Due to the fact that variances in the moisture content at different depths of the screen wall also need to be compared, the areas for the test were measured at the gray brick zone to ensure consistency.

## 3. Results and Discussion

### 3.1. Manufacturing Techniques Reflected by Composition and Textural Morphology

#### 3.1.1. Body

The SEM–EDS analysis revealed that all the glazed tile samples are made by siliceous paste (60.2–65.7 wt.% SiO_2_) and contain 15.0–18.7 wt.% of Al_2_O_3_. The pastes have relatively high components of CaO (7.9 wt.% on average), Na_2_O (2.3 wt.% on average), K_2_O (2.8 wt.% on average) and MgO (2.9 wt.% on average) as a fluxing agent. Additionally, 4.7 wt.% FeO content on average was observed, which explains the samples’ red hue fired in an oxide atmosphere. No significant differences in the major chemical compositions of glaze tile pastes could be observed between those used in the Confucian Temple screen walls and the Town God’s Temple screen walls. The same applied to the glazed tile pastes made in different periods. The chemical compositions of loess-based north Chinese ceramics and Chinese loess were also listed in Table 2. The paste composition characteristic of these glazed tiles is similar to that of loess-based glazed tiles of the Palace Museum and the loess of North China, which has the characteristics of having low SiO_2_ and Al_2_O_3_ content with a high content of fluxing oxides such as CaO and Na_2_O. This implies that the glazed tiles in this study are possibly also made by loess. Small differences in chemical composition could be found between the loess and tile pastes because the loess used for the tile pastes was prepared by sorting and elutriation. It also needs to be pointed out that the chemical composition of some early period loess-based north Chinese ceramics is different from that of the glazed tiles in this study, especially for their lower content of CaO. This demonstrates that the kind of loess used to make Hancheng glazed tiles is characterized by its Ca-rich minerals.

**Figure 6:**
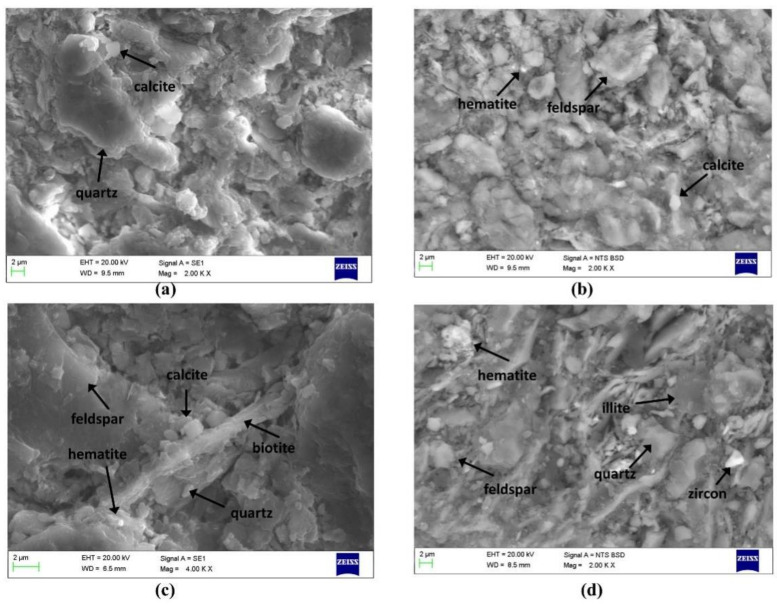
SEM backscattered electron mode images representative of inclusions of sample paste. (**a**) C-1; (**b**) C-8; (**c**) T-1 and (**d**) T-2.

Some inclusions of quartz, K-feldspar, calcite and hematite could be found in sample paste by SEM–SDS analysis, and sometimes illite, biotite and zircon also could be observed (the SEM images of sample C-1, C-8, T-1 and T-2 can be seen in Figure 6). The mineral phases of the paste samples identified by XRD have been summarized in Table 3; quartz (SiO_2_), K-feldspar (K_2_O·Al_2_O_3_·6SiO_2_), plagioclase (Na(AlSi_3_0_8_)–Ca(Al_2_Si_2_0_8_)) and hematite (Fe_2_O_3_) were observed in all the samples studied, and K-feldspar, plagioclase and hematite should be the main sources of Na_2_O, K_2_O and Fe_2_O_3_ in these pastes, respectively. This mineralogy, consistent with the SEM–EDS analyses, is indicative of common loess, which is mainly composed of non-clay minerals including quartz, K-felspar, plagioclase and calcite as well as clay minerals [14].

One significant difference between the paste samples was identified. Mullite was detected in most samples, while illite was found in five samples with no mullite detected. The presence of mullite in the paste samples indicates a relatively high firing temperature of at least 1000 °C, since this phase starts nucleation at a range of 1000–1100 °C from the calcium silicates in the matrix [15,16]. However, the paste samples that have mullite also contain dolomite and/or calcite, which usually do not exist above 900 °C [17]. A possible reason for this contradiction might be understood by the following explanation. Due to the influence of many factors, such as the poor control of firing atmosphere in ancient kilns, ancient glazed tiles might be heated unevenly in the firing process. The existence of mullite in tile paste at least implies that the local firing temperature for these paste samples may reach more than ca. 1000 °C. Calcite and dolomite in clay might decompose and produce CaO and MgO during high-temperature firing (above ca. 900 °C). Then CaO and MgO in tile paste might react with H_2_O and CO_2_ when the glazed tiles are exposed to outer conditions long-term. Finally, small amounts of CaCO_3_ (calcite) and CaMg (CO_3_)_2_ (dolomite) are formed. In contrast, the paste samples C5, T1, T2, T3 and T4, which have the mineral illite, might have been fired at a relatively low temperature, below 1000 °C [16]. This indicates that these architectural glazed tiles might have been produced at the firing temperature of ca. 900–1000 °C (this is only a tentative inference, and further analysis of sintering temperature is needed). They were produced in batches and at different times, or made in a different workshop, and the firing temperature of the different batches fluctuated. However, the difference in firing temperature is not reflected in the chronological sequence because the tiles decorated on the South screen wall and the screen wall of Biping Gate, both made in 1616 AD, seem to have been produced by different firing temperatures.

Both the chemical composition and mineral phase results of the tile samples demonstrate that all the tile pastes in this study were highly possibly made by local loess. However, according to published studies on the Chinese architectural glazed tiles used as building materials in the roof, the majority of the tile pastes were made from local clay or porcelain stone [18]. So far, it has been solely found that the paste of turquoise glazed tiles from the Beijing Palace Museum was produced from loess [13]. Loess has been selected as a raw material for ceramic production in North China for a significantly long period due to its good plasticity. For example, it was used in the Neolithic pottery of the Banpo Site and the terracotta sculptures depicting the armies of Qin Shi Huang, both located in the Shaanxi Province [12].

#### 3.1.2. Glaze

**Figure 7:**
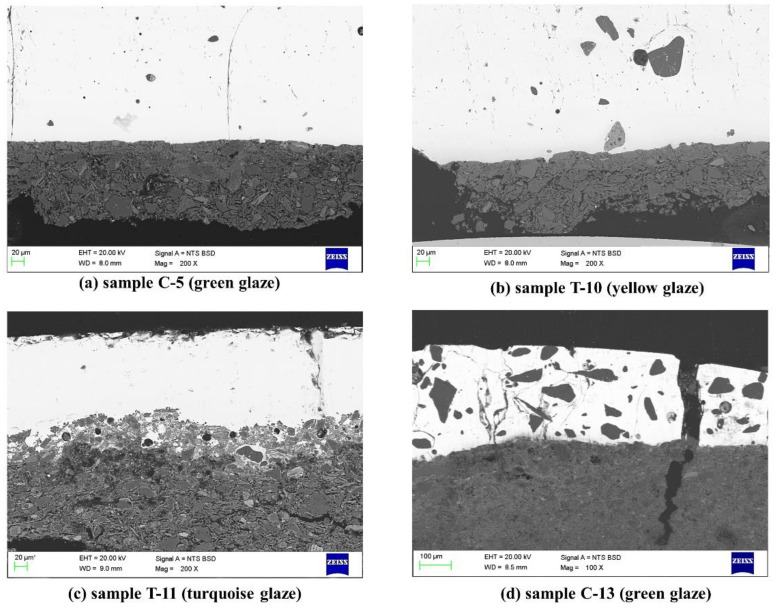
SEM backscattered electron mode images representative of glazed tile samples. (**a**–**c**) Glaze with a small number of quartz inclusions; (**d**) glaze with a large number of quartz particles.

Fifteen samples were observed by SEM backscattered electron mode images. The thickness of the glaze ranges from around 200 to 600 μm. The majority of the samples (13 of 15 samples) were found to contain a small number of quartz inclusions in the glaze as shown in Figure 7a–c, while two samples contain a large number of quartz particles in the glaze, as shown in Figure 7d. The quartz inclusions show variable sizes, between ca. 5 and 150 μm, and variable shapes: irregular, rectangular and oval. Additionally, bubbles of different sizes were also observed in the glaze. The sizes of bubbles are between ca. 5 and 50 μm in diameter. The quartz inclusions and bubbles, especially those of a large size, increase the opacity of the glaze and also contribute to the cracking and shedding of the glaze.

Images obtained by SEM also show that most of the samples in this study have almost no body-glaze interface (similar to the samples in Figure 7a,b,d) or a very clear and thin interface (similar to the sample in Figure 7c). This indicates that the glazed tiles were likely produced by a double-firing process. In specific, during the first time firing of the tile paste itself, most of the paste raw materials-loess form a relatively stable phase at high temperature. Then when the glaze suspension is applied on the already biscuit-fired body for the second time firing, a fewer concentration of diffused elements between glaze and paste is formed, and then leads to a less obvious or thinner glaze-body interface [19].

As highlighted in Table 4, the glaze of the tile samples is mainly comprised of SiO_2_, Al_2_O_3_, PbO, K_2_O, Na_2_O, MgO, CaO, CuO and FeO. Among them, SiO_2_ and Al_2_O_3_ are indispensable components in all types of ceramic glaze. They react with flux oxides at a high temperature to form the glaze. PbO, K_2_O, Na_2_O, MgO and CaO are common flux oxides and are used in different types of glazes to reduce the firing temperature of glaze production. CuO and FeO are the main colorants for glazes.

Samples of the glazed tiles considered in this study can be classified into two subgroups, according to their color and chemical composition.

##### Green, Yellow and Brown Glazes

Table 4 highlighted that the glaze samples which have a green, yellow and brown colors contain similar levels of SiO_2_ (ranging from 28.3 to 35.5 wt.% and 32.1 wt.% on average), Al_2_O_3_ (ranging from 0.9 to 2.7 wt.% and 1.9 wt.% on average), PbO (ranging from 54.5 to 62.8 wt.% and 58.9 wt.% on average), K_2_O+Na_2_O (ranging from 0.7 to 2.1 wt.% and 1.5 wt.% on average) and MgO + CaO (ranging from 0.8 to 2.5 wt.% and 1.5 wt.% on average). This indicates that all these glazes are a SiO_2_-Al_2_O_3_-PbO system, with lead oxide as the primary flux in the glaze. This corresponds to the results of previous studies which demonstrated that architectural tiles are normally decorated with a typically high-lead glaze, such as the glazed tiles which decorate the palaces and halls in the Beijing Palace Museum [20].

The analysis revealed that the green glaze samples have the largest copper oxide (CuO) component, ranging from 2.3 to 5.5 wt.%. This reveals that that CuO is the coloring agent. The yellow and brown glaze samples have a relatively high iron oxide (FeO) content ranging from 2.2 to 5.0 wt.%. This infers that Fe_2_O_3_ was used as the coloring agent for the yellow and brown glaze. In Chinese low-fired lead glaze, both the brown and yellow glaze are colored by iron oxide and fired in oxide atmospheres. In some cases, brown glaze is named as dark yellow glaze, and sometimes brown glaze and yellow glaze are called as dark amber and light amber, respectively. The color tones of the glazes are affected by the colorant content, firing atmosphere, ratios of Fe^3+^/Fe^2+^ and other comprehensive factors.

There are two historical records that mentioned the glaze recipe of architectural glazed tiles, and the details have been organized in Table 5. The chemical compositions of tile glazes in this study are basically consistent with the glaze formula recorded in ancient literature. The lithargite mentioned in the ancient glaze recipe provides the content of PbO in glaze. The Luohe stone and Maya Stone are both quartzite collected from different areas, which provide the SiO_2_ content in the glaze. They contain a high SiO_2_ content, generally higher than 90% and up to more than 98%. Copper powder and ochre are the coloring materials of the green glaze and the yellow glaze, respectively. However, if we ignore the impurities in the raw materials and only calculate the contents of SiO_2_, Al_2_O_3_, CuO and FeO (the results are shown in Table 5), it can be found that compared to the chemical compositions of green and yellow glazes analyzed in this study, the glaze recipe in historical records had a higher PbO content combined with a lower SiO_2_ content. This infers that the glaze recipe mentioned in historical records has higher PbO/SiO_2_ ratio (3:1) than that of glazes in this study (ranging from 1.6 to 2.2, and 1.8: 1 on average).

The lower PbO/SiO_2_ ratios in tile glazes studied might be understood by the following three explanations: (A). In the actual glazed tile production, the glaze-making formula may be slightly different in different periods and regions; (B). In the actual firing process of the glaze, lead oxide might volatilize to some extent; (C). Following hundreds of years of erosion, the lead oxide in the glaze may have been dissolved by precipitation. This could have then led to the reduction of the lead content.

##### Turquoise Glaze

**Figure 8:**
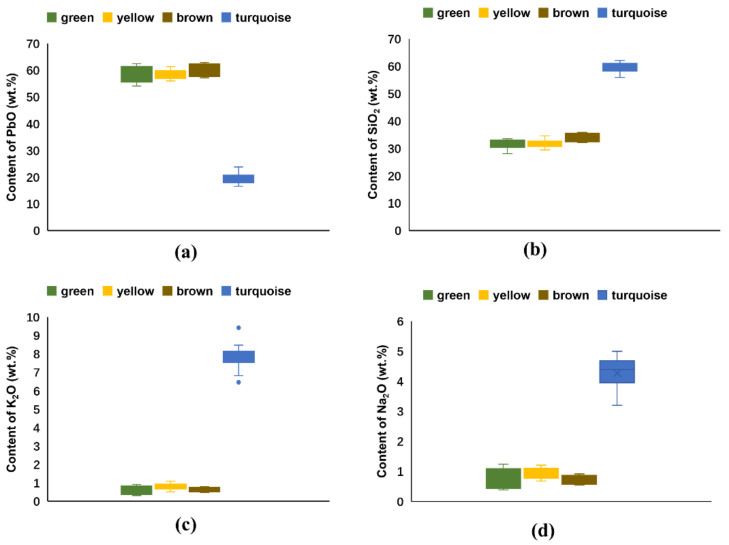
Plots of chemical compositions in glaze samples with different colors. (**a**) Content of PbO; (**b**) Content of SiO_2_; (**c**) Content of K_2_O and (**d**) Content of Na_2_O.

As Figure 8 highlights, in relation to the composition of the turquoise glazes, they are significantly different to the glaze samples of green, yellow and brown. Specifically, the turquoise glaze samples have lower contents of PbO (ranging from 16.7 to 24.0 wt.% and 19.5 wt.% on average) with higher contents of SiO_2_ (ranging from 56.3 to 62.6 wt.% and 60.0 wt.% on average), K_2_O (ranging from 6.5 to 9.5 wt.% and 7.9 wt.% on average) and Na_2_O (ranging from 3.2 to 5.0 wt.% and 4.3 wt.% on average). This indicates that the turquoise glaze is a lead-alkaline glaze, mainly fluxed by PbO, K_2_O and Na_2_O together. Additionally, CuO (ranging from 2.0 to 4.9 wt.% and 3.6 wt.% on average) is used as the coloring agent. Copper ions can appear peacock blue or peacock green in color in alkaline oxide flux.

In China, the turquoise glaze of the SiO_2_-PbO-alkaline system, which was originally used for building materials of imperial palaces and temples, can be traced back to the middle period of the Tang dynasty (618–907 AD). Since the Northern Song dynasty (960–1127 AD), turquoise glaze began to be used in Chinese ceramics and its SiO_2_-PbO-alkaline recipe became essential in the low-temperature glaze system of Chinese ceramics [21]. The famous Chinese glazes such as Fahua glazes and Cizhou polychrome alkaline glazes both have the SiO_2_-PbO-alkaline recipe. It needs to pointed out that the maturing temperature of SiO2-PbO-alkaline recipe is normally above 1000 °C, which is higher than any typical Chinese high-lead glaze so far discussed (900–1000 °C) [12,22]. The SiO_2_-PbO-alkaline is classified as a low-fired glaze in the history of Chinses glazes because it uses lead oxide as the main fluxing agent, which is totally different from the Chinese high-fired calcium glazes. The contents of alkaline (K_2_O and Na_2_O) in this recipe comes from nitrate and changes by different origins of the nitrate used. For example, the architectural turquoise-glazed tiles used in the Beijing Palace Museum [13] all have a similar glazing composition, of a SiO_2_-PbO-K_2_O system, whereas the glaze examined in this paper reveals that K_2_O and Na_2_O were combined to make the alkaline component. This indicates that distinct sources of nitrate were used in the turquoise glaze making of different areas.

### 3.2. Degradation Process

**Figure 9:**
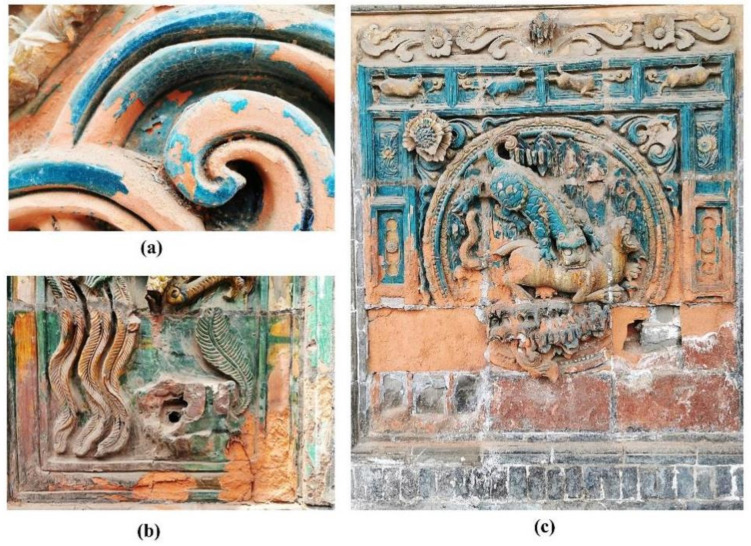
Decay images. (**a**,**b**) Glaze cracking and shedding; (**c**) Body salt efflorescence.

Field investigations found that due to long-term exposure to the open environment, multiple types of decay phenomena have occurred in these glazed tiles which decorate the screen walls. The glaze cracking and shedding and body salt efflorescence shown in Figure 9 are the most widespread and damaging types of decay.

#### 3.2.1. Glaze Cracking and Shedding

Field investigations found that cracking and shedding are the most common deterioration to occur to tile glazes. This is mainly affected by the lead glaze production process itself and the exposure to the outdoor environment.

For the glazing technique, the glaze-body fit is an important factor for the stability of the glazed tile. If the thermal contraction of the glaze is greater than that of the body, the glaze will be under tension and the tensile stresses can cause cracking or ‘crazing’ of the glaze surface [20]. The optimum situation of the glaze-body fit is that the contraction of the glaze should be around 5–15% less than that of the body [23]. As discussed in Section 3.1.2, the glazed tiles studied in this research are decorated by lead glaze that has undergone a double firing process. The content of SiO_2_ in the tile body is equivalent to that of PbO in the glaze, both of which are above 50 wt.% (for the turquoise glaze, the total content of PbO and SiO_2_ is higher than 50 wt.%). Lead oxide has a relatively high thermal expansion coefficient. In the firing range of 1–100 °C, the thermal expansion coefficient of PbO is 4.2 × 10^−7^/°C, while that of SiO_2_ is relatively low, at 0.8 × 10^−7^/°C [24]. This means that for the lead glaze recipe, theoretically, the thermal expansion coefficient of the glaze should typically be higher than that of the body. Besides, according to Tite et al. (1998) [19], thermal expansion coefficients for typical high-lead glazes vary from 5 to 7 × 10^−6^/°C. In comparison, the expansion coefficients for non-calcareous earthenware bodies in the 0–500 °C range vary from 3 to 5 × 10^−6^/°C and from 5 to 7 × 10^−6^/°C for those made from calcareous clays (i.e., clays containing typically 15–25% of well-dispersed CaO). This means that in this study, theoretically, the thermal expansion coefficients of lead glazes might be similar or higher than their bodies (with 5.2–10.2% contents of CaO). Additionally, this theory that lead glazes have higher thermal expansion coefficients compared to their pastes has also been evidenced through the published data analysis of a range of high-lead glazed pottery [20] as well as via the glazed tiles which decorate the Beijing Palace Museum [24]. Consequently, the primary internal cause of the glaze-cracking process is the tensile stress of the lead glaze due to its higher expansion coefficient. Additionally, for those glazes with quartz inclusions and bubbles, the heterogeneity of the glaze also contributes to the cracking of the glaze.

The architectural glazed tiles are exposed to the atmospheric environment, which is more related to the problems of glazed tile conservation. The drastic changes with temperature and humidity (such as high temperature and torrential rain) can further lead to the glaze cracking because of the higher expansion coefficient of the glaze. When the tensile stresses difference at the joint of the tile body and glaze exceeds the limit, the glaze layer will even peel off. The glaze cracks not only weaken the strength of the glaze layer, but also allow the water (and salt) migration and crystallization to occur in both the tile glaze and body, which further leads to the glaze crazing and shedding.

#### 3.2.2. Body Salt Efflorescence

Body salt efflorescent is another widespread phenomenon that occurs to the glazed tile decorations on the screen wall, and this disease normally occurs in a range of about 1–2 m from the ground. Although it is not discussed in this paper, the main body of the screen wall—the gray bricks—also suffer from the salt efflorescence disease in a similar area. As shown in Figure 9c, the tile glaze peels off, and the tile body looks loose. Additionally, some glazed tiles that seem to have intact glaze and are well preserved have also begun to undergo salt efflorescence.

**Figure 10:**
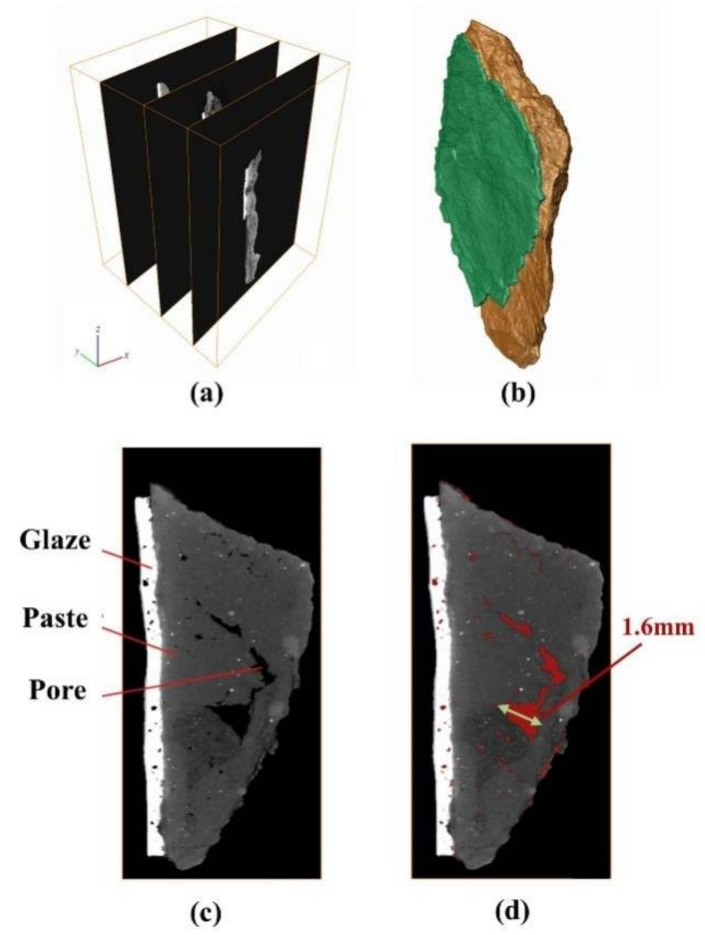
(**a**) The raw CT scanning grayscale slice sequence; (**b**) The 3D reconstruction model; (**c**) The middle cross-section image in grayscale; (**d**) The extracted pore area of the middle cross-section. (Note: sample T-15; the green part of 3D reconstruction model is the glaze layer of the sample, the pore area is rendered in red).

The sample T-15 with intact glaze but weathered-looking paste has been scanned by a micro-CT scanner. Sequences of grayscale images were reconstructed into a 3D image, shown in Figure 10a,b. In the grayscale sliced image, the gray level is related to material density, as the brighter part is denser and the darkest part is the void. The image of the middle cross-section inside the sample is presented and rendered in Figure 10c,d. It can be seen that there are big and even connected pores with irregular shapes in the paste layer beneath the glaze layer, the biggest one showing a size of 1.6 mm. For those small holes in the paste, they either might be formed in the process of tile production or might be formed by weathering. However, for those connected holes with such big sizes above 1 mm, they demonstrate that the tile paste has suffered from salt efflorescent decay and has led to big pores in the paste, although the glaze layer of the sample is well-preserved temporarily.

**Figure 11:**
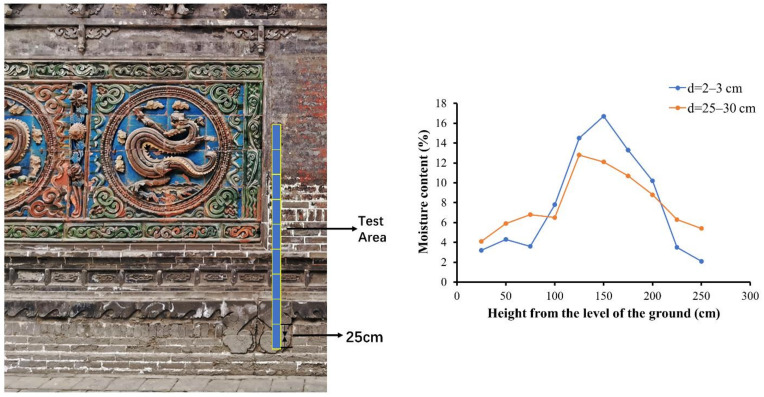
The moisture contents at different heights of the Five-Dragon screen wall. (d means the depth from the surface).

To further explore the causes of the body salt efflorescent process, the moisture contents at different heights of five screen walls have been tested with non-destructive moisture measurements. The result of the Five-Dragon screen wall is shown in Figure 11. This demonstrates that the moisture content of the wall is clearly higher in the height range of approximately 1–2 m, which basically overlaps with the areas which have suffered from serious salt efflorescent decay. Additionally, the moisture content of the surface area (d = 2–3 cm) is evidently higher than that of the interior area (d = 25–30 cm).

The samples of solute salt powder collected from the Five-Dragon screen wall and screen wall of Biping Gate were analyzed by XRD. The results show that the samples of solute salt powder mainly contain quartz (SiO_2_), gypsum (CaSO_4_) and hexahydrite (MgSO_4_·6H_2_O). Since the sampling process inevitably brought in a small amount of tile paste, the tile pastes should be the source of SiO_2_ analyzed by XRD. In addition, CaSO_4_ and MgSO_4_·6H_2_O were detected and should be the main soluble salts.

This infers that the salt efflorescence of the tile body (including the gray bricks) is mainly due to the fact that the groundwater carrying the soluble salts is drawn into the screen wall through a capillary process. Then under the joint influence of thermal evaporation and capillarity, capillary water (and soluble salts) rises continuously and gradually migrates to the wall surface, finally concentrating in the height with the range of 1–2 m from the ground. When soluble salts crystallize, their volume will expand. Additionally, with the cycle of air temperature and relative humidity, the soluble salts carried by the migrated water undergo repeated crystallization. This means that when exposed to outer conditions for a long term, the soluble salts in the tile bodies are repeatedly dissolved, expanded and crystallized, which results in the looseness of the tile paste and even the formation of holes. Additionally, the infiltration of the rainwater (and soluble salt) into the tile body along the glaze cracks is another reason for the salt efflorescence. Eventually, the salt efflorescence breaks down the tile body, ultimately causing the glaze to be lost and even resulting in the shedding of the entire glazed tile.

### 3.3. Conservation Suggestions

#### 3.3.1. Water Proofing

As discussed in Section 3.2.2, the salt efflorescence of the tile body (including the gray bricks) caused by groundwater (solute salt) can heavily weaken the stability of the screen wall and its glazed tile decoration. Therefore, protecting screen walls from dampness and moisture is the primary task. Specifically, the wall foundation should be exposed for water proofing treatment. The organic silicon resin materials with excellent hydrophobicity can be selected to cover and penetrate the exposed basement wall. Then the screen wall foundation should be backfilled with a mixture of lime and local soil, which is the traditional waterproof building material commonly used in China. This method can effectively prevent the foundations from absorbing groundwater. Additionally, organic silicon resin materials also must be grouted into the screen wall at a height of approximately 30 cm above the ground in order to prevent the wall root from absorbing rainwater and groundwater.

#### 3.3.2. Desalination

Desalination treatment should be carried out on the glazed tiles and bricks of the screen walls which have deteriorated through soluble salt crystallization. In this study, desalination should be mainly applied to glazed tiles and bricks at a height of 1–2 m from the ground. Paper pulp and sepiolite specially used for cultural relics protection can be poulticed in the surface of the glazed tiles and bricks. In doing so, the soluble salt inside the glazed tiles and bricks can migrate to the externally applied material. This desalination process needs to be repeated until the surface salt content is constant.

#### 3.3.3. Reproduction

For the areas where the glazed tiles or glaze layers have shed, we can reproduce the glazed tiles to reestablish the visual integrity of the glazed tile decoration of the screen walls on a trial basis. Besides, for the glazed tiles which have decayed seriously, it is also an effective preservation measure to move them to the museum for further protection and display and using reproduced ones to replace them in situ. The raw materials and techniques of making these Hancheng glazed tiles have been discussed in this study. Therefore, the glazed tiles can be reproduced using traditional methods. It is important that the replicas should be lightly distinguished from the original glazed tiles in both color tone and gloss.

## 4. Conclusions

This research has furthered the understanding of the architectural glazed tiles which decorate ancient Chinese screen walls based on scientific study. The tile pastes of the screen walls from the Hancheng Confucian Temple and the Town God’s Temple in Shaanxi Province are potentially made from loess, which is not a common raw material used to produce glazed tiles. Tile glazes can be classified into two subgroups according to different colors and glaze recipes. The tile glazes with green, yellow and brown colors are typical high-lead glazes with a glaze recipe of SiO_2_-Al_2_O_3_-PbO. On the other side the turquoise glazes are produced by a glaze recipe of SiO_2_-PbO-alkaline (K_2_O and Na_2_O). The chemical compositions of tile glazes in this study are basically consistent with the glaze formula of architectural glazed tiles recorded in ancient literature. The microstructures show that most of the glazed tile samples have almost no paste-glaze interface, which infers that they are produced by a double-firing process. The microstructures and chemical compositions of the glazed tiles between the Ming and Qing dynasties were consistent. This indicates that the raw material selection and relevant manufacturing techniques had been optimized and kept unchanged to produce these glazed tiles in Hancheng city for a long period.

The most widespread and damaging degradation of these glazed tiles are glaze cracking and shedding and body salt efflorescence. For the deterioration of glaze cracking and shedding, the internal cause is the tensile stress of the glaze due to its higher expansion coefficient compared to tile paste. Besides, the drastic environmental changes such as high temperature and torrential rain, as well as the water (and salt) migration and crystallization within the glazed tiles, are the external factor. The salt efflorescence of the tile body normally occurs in the range of about 1–2 m from the ground. The main cause is that the groundwater carrying soluble salt is drawn into the screen wall through a capillary process, and the soluble salts undergo repeated crystallization. Therefore, preventing the permeation of water into the glazed tiles and desalination are the most urgent tasks to protect the glazed tiles which decorate screen walls from further degradation.

This research enables us to better understand the raw materials and technological choices used to produce the glazed tiles which decorate ancient Chinese screen walls and also provide insights into the causes for their key degradation processes. All these have laid a solid foundation to propose effective preservation and restoration treatments.

## Figures and Tables

**Table 1:** Sample information.

Sample No.	Location	Production Year	Glaze Color	Sample No.	Location	Production Year	Glaze Color
Glazed tile samples from the Hancheng Confucian Temple				Glazed tile samples from the Hancheng Town God’ s Temple			
**C-1**	Five-Dragon screen wall	within the Wanli Period of the Ming Dynasty (1573–1620 AD)	green	**T-1**	South screen wall	the 44th year of the Wanli Period of the Ming Dynasty (1616 AD)	turquoise
**C-2**			green and yellow	**T-2**			turquoise
**C-3**			turquoise	**T-3**			yellow
**C-4**			brown	**T-4**			yellow
**C-5**	East screen wall of Lingxing Gate	the 18th year of the Chenghua Period of the Ming Dynasty (1482 AD)	green	**T-5**	West screen wall of Biping Gate	the 44th year of the Wanli Period of the Ming Dynasty (1616 AD)	turquoise
**C-6**			green and yellow	**T-6**			turquoise
**C-7**			yellow	**T-7**			yellow
**C-8**	East screen wall of Halberd Gate	the 18th year of the Chenghua Period of the Ming Dynasty (1482 AD)	green	**T-8**			turquoise
**C-9**			turquoise	**T-9**	East screen wall of Biping Gate	the 44th year of the Wanli Period of the Ming Dynasty (1616 AD)	yellow
**C-10**			turquoise	**T-10**			yellow
**C-11**			brown	**T-11**			turquoise
**C-12**	West screen wall of Halberd Gate	the 18th year of the Chenghua Period of the Ming Dynasty (1482 AD)	yellow	**T-12**	Screen walls of Xizhi Gate	within the Kangxi Period of the Qing Dynasty (1662–1722 AD)	yellow
**C-13**			green and yellow	**T-13**			green and brown
**C-14**			green and yellow	**T-14**			green
**C-15**			yellow and turquoise	**T-15**			green
**C-16**			brown	**T-16**	East screen wall of Biping Gate	the 44th year of the Wanli Period of the Ming Dynasty (1616 AD)	turquoise
**C-17**			turquoise				

**Table 2:** Major chemical compositions of the body of Hancheng glazed tile samples and loess from North China (wt.%). The data of loess from North China is taken from Wu et al. (1996) [11]; loess-based north Chinese ceramics is from Wood (2011, 197) [12]; glazed tiles of the Palace Museum is from Kang et al. (2018) [13] and the N means number of samples.

Sample No.	SiO_2_	Al_2_O_3_	Na_2_O	K_2_O	MgO	CaO	FeO
Glazed tile samples from the Hancheng Confucian Temple							
**C-1**	60.2	18.7	1.8	4.1	3.1	5.9	6.1
**C-2**	61.7	18.1	1.9	3.3	3.4	5.2	6.3
**C-3**	61.9	18.5	2.5	2.8	3.0	5.8	5.4
**C-4**	62.5	17.2	2.7	3.1	3.0	8.1	3.5
**C-5**	62.4	16.5	2.1	2.9	2.6	8.3	5.2
**C-6**	64.5	16.4	3.2	2.4	2.5	6.8	4.2
**C-7**	61.3	15.0	2.9	4.1	2.8	8.1	6.0
**C-8**	64.2	17.4	1.4	2.4	2.5	8.8	3.3
**C-9**	62.5	17.0	2.3	2.4	3.0	8.7	4.0
**C-10**	60.9	15.9	2.1	2.2	2.9	10.5	5.3
**Glazed tile samples from the Hancheng Town God’ s Temple**							
**T-1**	61.8	16.6	1.4	2.8	2.2	9.3	5.9
**T-2**	60.9	17.3	2.6	2.7	3.6	7.5	5.5
**T-3**	61.8	16.9	2.4	2.2	3.5	10.2	3.2
**T-4**	63.4	17.7	1.9	1.9	2.6	9.8	2.9
**T-5**	63.5	17.8	1.7	2.5	2.9	7.1	4.6
**T-6**	61.8	16.7	2.9	3.1	2.9	8.3	4.0
**T-7**	62.6	14.6	3.2	3.5	2.7	9.6	3.7
**T-8**	62.9	15.8	2.8	3.4	2.3	7.4	5.3
**T-9**	61.7	16.0	2.7	2.7	4.0	6.8	6.0
**T-10**	65.7	17.7	2.5	2.3	2.5	5.4	4.0
**T-11**	64.5	16.1	2.6	1.8	3.0	7.7	4.3
**T-12**	65.7	16.5	2.3	2.1	2.4	7.3	3.7
**T-13**	62.1	16.9	1.7	4.0	3.0	8.0	4.4
**Loess of North China (chemical compositions on average) [11]**							
**Sandy loess (N = 23)**	69.3	12.9	2.2	2.5	2.6	7.8	2.8
**Loess (N = 97)**	66.3	13.7	1.9	2.6	2.7	9.3	3.3
**Clay loess (N = 27)**	65.6	13.9	1.8	2.6	2.7	10.1	3.4
**Analysis of loess-based North Chinese ceramics [12]**							
**Qin terracotta warrior, Lishan**	66.3	16.6	2.0	3.3	2.3	2.0	6.1
**Qin terracotta horse, Lishan**	63.2	15.9	1.6	2.9	2.1	2.6	6.1
**Han dynasty funerary jar**	65.8	15.8	1.6	3.3	2.1	2.1	5.2
**Shang dynasty earthenware pipe**	66.5	16.9	1.3	3.0	2.0	2.8	6.5
**Yuan dynasty glazed tiles (N = 8), the Palace Museum [13]**	62.1	16.8	2.2	2.6	2.6	8.2	4.6

**Table 3:** Mineral composition of the body of Hancheng glazed tile samples.

Sample No.	Location	Mineral Composition
**C-1**	Five-Dragon screen wall (1573–1620AD)	quartz, K-feldspar, plagioclase, mullite, calcite, dolomite, hematite
**C-2**		quartz, K-feldspar, plagioclase, gypsum, mullite, hematite
**C-3**		quartz, K-feldspar, plagioclase, mullite, dolomite, hematite
**C-5**	East screen wall of Lingxing Gate (1482AD)	quartz, K-feldspar, plagioclase, calcite, illite, hematite
**C-6**		quartz, K-feldspar, plagioclase, dolomite, mullite, hematite
**C-8**	East screen wall of Halberd Gate (1482AD)	quartz, K-feldspar, plagioclase, mullite, calcite, dolomite, hematite
**C-9**		quartz, K-feldspar, plagioclase, mullite, gypsum, calcite, dolomite, hematite
**C-10**		quartz, K-feldspar, plagioclase, gypsum, mullite, hematite
**T-1**	South screen wall (1616AD)	quartz, K-feldspar, plagioclase, calcite, illite, hematite
**T-2**		quartz, K-feldspar, plagioclase, calcite, illite, dolomite, hematite
**T-3**		quartz, K-feldspar, plagioclase, gypsum, illite, hematite
**T-4**		quartz, K-feldspar, plagioclase, calcite, illite, hematite
**T-5**	West screen wall of Biping Gate (1616AD)	quartz, K-feldspar, plagioclase, mullite, dolomite, hematite
**T-8**		quartz, K-feldspar, plagioclase, dolomite, mullite, hematite
**T-9**	East screen wall of Biping Gate (1616AD)	quartz, K-feldspar, plagioclase, dolomite, mullite, hematite
**T-10**		quartz, K-feldspar, plagioclase, mullite, hematite
**T-12**	Screen walls of Xizhi Gate (1662–1722AD)	quartz, K-feldspar, plagioclase, mullite, calcite, hematite
**T-13**		quartz, K-feldspar, plagioclase, mullite, calcite, hematite

**Table 4:** Major chemical compositions of the glaze of Hancheng glazed tile samples (wt.%, b.d. means below the detection limits).

Sample No.	Glaze Colour	PbO	SiO_2_	Al_2_O_3_	FeO	CuO	Na_2_O	K_2_O	CaO	MgO	TiO_2_
**C-1G**	Green	62.7	28.3	1.9	0.8	2.6	1.1	0.6	1.2	0.8	b.d.
**C-2G**		59.0	31.6	1.8	0.5	3.7	1.0	0.3	1.3	0.3	0.2
**C-5G**		61.3	30.1	2.3	0.5	2.9	0.4	0.3	0.9	0.9	0.1
**C-6G**		62.8	30.6	1.8	0.6	2.3	0.4	0.4	0.8	0.2	0.1
**C-8G**		56.4	33.2	2.7	0.7	4.3	0.5	0.9	0.5	0.8	b.d.
**C-13G**		58.3	32.3	2.0	0.5	4.6	0.4	0.9	0.5	0.5	0.2
**C-14G**		57.6	31.7	2.1	0.7	3.7	0.7	0.7	1.3	0.9	0.2
**T-13G**		54.5	33.8	1.3	0.9	5.5	1.1	0.8	1.3	0.6	0.1
**T-14G**		55.2	33.7	2.0	0.9	4.5	1.3	0.8	0.9	0.6	0.2
**T-15G**		60.1	30.6	2.2	0.6	3.1	0.9	0.8	1.0	0.4	b.d.
**C-2Y**	Yellow	58.5	32.1	2.2	3.1	0.2	1.1	0.7	1.1	0.5	0.2
**C-6Y**		57.0	33.2	2.5	3.0	b.d.	1.1	0.8	1.6	0.9	b.d.
**C-7Y**		55.9	33.6	2.7	2.7	b.d.	1.2	0.9	1.6	0.7	0.2
**C-12Y**		59.4	32.2	1.7	3.3	b.d.	1.0	0.9	0.9	0.4	0.2
**C-13Y**		60.6	30.7	1.5	4.0	0.1	1.0	0.8	0.8	0.5	b.d.
**C-14Y**		57.3	34.9	0.9	3.7	b.d.	0.9	1.1	0.8	0.4	b.d.
**C-15Y**		61.8	31.1	0.9	3.3	b.d.	0.8	0.9	0.7	0.6	b.d.
**T-3Y**		58.5	31.8	1.7	4.6	b.d.	0.7	0.8	0.8	0.7	0.1
**T-4Y**		59.9	31.2	2.0	4.5	0.1	0.7	0.5	0.9	0.2	b.d.
**T-7Y**		59.7	30.6	2.0	4.6	b.d.	0.7	0.7	1.1	0.4	0.2
**T-9Y**		57.2	32.6	1.8	5.0	b.d.	0.9	0.7	1.3	0.5	0.2
**T-10Y**		60.3	29.6	1.8	4.0	b.d.	0.9	1.0	1.8	0.6	b.d.
**T-12Y**		59.4	32.1	2.4	2.6	0.1	1.2	0.7	0.6	0.7	b.d.
**C-4B**	Brown	58.9	32.2	2.2	4.2	b.d.	0.9	0.8	0.6	0.3	b.d.
**C-11B**		60.8	31.2	1.5	4.3	b.d.	0.6	0.6	0.6	0.5	b.d.
**C-16B**		58.3	35.5	1.6	2.4	b.d.	0.7	0.7	0.5	0.3	b.d.
**T-13B**		58.8	35.4	1.6	2.2	b.d.	0.6	0.5	0.7	0.2	b.d.
**C-3T**	Turquoise	18.6	62.6	1.5	0.6	2.0	4.7	7.7	0.9	0.9	b.d.
**C-9T**		16.7	62.1	2.0	0.3	2.3	4.5	9.5	1.4	0.9	b.d.
**C-10T**		17.7	62.3	1.1	0.7	3.4	4.8	8.5	0.8	0.4	b.d.
**C-15T**		18.8	60.3	2.6	0.7	2.6	4.1	8.2	0.9	1.4	0.3
**C-17T**		21.2	59.3	1.2	0.4	3.8	4.2	7.7	0.9	0.7	0.2
**T-1T**		22.0	56.3	1.5	0.9	4.7	3.2	7.7	1.5	1.8	0.1
**T-2T**		24.0	56.7	1.8	0.6	4.4	3.9	6.9	0.9	0.6	0.2
**T-5T**		19.9	59.0	2.1	0.9	4.1	3.6	8.2	0.9	1.0	b.d.
**T-6T**		18.4	60.6	2.0	0.3	4.3	4.1	7.9	1.1	1.1	b.d.
**T-8T**		17.4	60.1	1.6	1.0	4.9	4.9	7.9	0.8	1.0	0.1
**T-11T**		19.8	60.2	1.3	0.7	3.4	4.6	7.8	1.0	0.5	0.2
**T-16T**		19.4	60.4	2.3	0.8	3.2	5.0	6.5	0.9	1.1	0.2

**Table 5:** The glaze formula of glazed tile production in historical records.

Information of Historical Record		Glaze Recipe of Glazed Tile				
Name	Description	PbO	SiO_2_	FeO	CuO	PbO:SiO_2_
Yingzao Fashi	Published in 1103 AD of the Song Dynasty The oldest technical treatise on architecture and craftmanship found in China	Lithargite 1500 g 69.9 wt.%	Luohe stone (a kind of quartzite) 500 g 23.3 wt.%	/	Copper powder 150 g 7.0 wt.%	3:1
Gongbuchangku Xuzhi	Published in 1615 AD of the Ming Dynasty It records the rules and management system of construction engineering in the Ming Dynasty	Lithargite 153 kg 73.6 wt.%	Maya stone (a kind of quartzite) 51 kg 24.5 wt.%	Ocher(a kind of hematite) 4 kg 1.9 wt.%	/	3:1
